# Global trend analysis of diabetes mellitus incidence, mortality, and mortality-to-incidence ratio from 1990 to 2019

**DOI:** 10.1038/s41598-023-49249-0

**Published:** 2023-12-11

**Authors:** Mohammadreza Balooch Hasankhani, Hossein Mirzaei, Ali Karamoozian

**Affiliations:** 1 Modeling in Health Research Center, Institute for Futures Studies in Health, Kerman University of Medical Sciences, Kerman, Iran; 2 HIV/STI Surveillance Research Center, WHO Collaborating Center for HIV Surveillance, Institute for Futures Studies in Health, Kerman University of Medical Sciences, Kerman, Iran; 3 Department of Biostatistics and Epidemiology, School of Public Health, Kerman University of Medical Sciences, Kerman, Iran

**Keywords:** Diabetes, Health policy

## Abstract

Diabetes Mellitus (DM) is a common chronic disease and a public health challenge worldwide. The present study aimed to analyze the trend in DM incidence, mortality, and mortality-to-incidence ratio (MIR) world over 30 years. The age-standardized DM incidence and mortality rates for both genders and different countries of the world from 1990 to 2019 were extracted from the Global Burden of Disease (GBD) study and the Human Development Index (HDI) was obtained for all countries from the United Nations Development Program (UNDP) database. The mean trends for the indicators for developing countries, developed countries, and all countries were evaluated using Generalized Estimating Equations (GEE) and the spline model. The estimates showed that the global mean trend of DM incidence from 1990 to 2019 followed an upward trend with an annual increase of 3.73 cases per 100,000 people. The global mean of DM mortality followed an upward trend with an annual increase of 0.43 cases until 2005 followed by a downward trend after this year with an annual decrease of 0.14 and the global mean MIR followed a downward trend during the same period with an annual decrease of 0.001 per 100,000 people. Besides, the mean incidence of DM in developing countries followed an upward trend similar to the trend in developed countries. Additionally, the mean mortality rate due to DM in developing countries increased with a slope of 0.62 until 2005 and then decreased with a slope of 0.02, and the mean MIR trend in the developed and developing countries showed a downward trend. Thus, developed countries followed a relatively faster decrease in MIR than developing countries.The data from the present study show the increased incidence of DM has made it one of the most important health concerns in the world, and if this issue is not addressed, this disease can cause more concerns for communities in the coming years. This being so, more DM prevention and control programs need to be put into practice.

## Introduction

Diabetes mellitus (DM) is a global public health problem that imposes a heavy global burden on public health and is associated with socio-economic consequences^1^. Various studies have shown that the incidence of DM has increased in recent decades^2,3^. It is predicted that the number of people with DM will increase from 171 million in 2000 to 300 million by 2025 and 366 million by 2030^4–6^. The World Health Organization (WHO) report on DM mortality showed that the mortality rate due to DM increased by 3% from 2000 to 2019^7^, and DM is the leading cause of death of 1.5 million people in 2019^8^.

DM can lead to complications in different body organs and tissues and aggravate other disorders and complications, leading to a heavy economic burden. The mean medical costs for people with DM are estimated to be 2.3 times higher than the medical costs of people without DM. The American Diabetes Association (ADA) reported that the cost of diagnosed DM in 2017 was $327.2 billion. Given the growing economic costs and the social burden that DM and its complications impose on societies^9^, there is a need for further research on DM in all countries.

Generally, the increasing incidence of DM in different regions is different due to several factors such as lifestyle, eating habits, access to health care, etc. Furthermore, the prevalence of obesity is increasing all over the world^10^ due to high-calorie diets and sedentary lifestyles. Thus, the increased prevalence of obesity along with population aging is one of the main reasons behind the increased prevalence of DM^11^. Another factor affecting variations in the prevalence of DM in different countries can be the development rate of the countries. According to the WHO, the prevalence of DM in low- and middle-income countries has increased at a faster rate than in high-income countries^7^. Moreover, IDF Diabetes Atlas (2021) reported that three out of every four adults with DM live in low and middle-income countries^12^. The Human Development Index (HDI) is one of the important measures of mean achievement, living conditions, and human development in different countries. Thus, it is necessary to compare developing and developed countries in terms of the prevalence of diabetics to help policymakers in controlling the process of this disease.

Most of the epidemiological studies conducted on the incidence and mortality rate of DM have used descriptive or cross-sectional designs. Furthermore, no study has yet addressed the effects of the development and geographical location on the incidence, mortality, and MIR of diabetics. For example, in recent years, several epidemiological studies have investigated the incidence and mortality of DM in different parts of the world. Jinli Liu et al. reported that the age-standardized incidence of diabetics worldwide increased from 234 in 1990 to 285 cases per 100,000 in 2017^13^. Xiling Lin et al. also showed that the age-standardized mortality rate worldwide increased from 15.7 to 17.5 per 100,000 people from 1990 to 2017^14^. Thus, a suitable statistical technique with a high degree of accuracy needs to be used to describe and compare this diversity in the incidence of diabetics in different regions from 1990 to 2019. To this end, using longitudinal data, the present study aimed to examine the DM incidence and mortality rates worldwide and specify the impact of development on the variations in the DM incidence and mortality rates in different regions of the world. The insights from this study can be used by international policymaking support centers active in the field of diabetics to bridge the existing gaps.

## Methods

### Data sources

In this study, the data on the age-standardized DM incidence and mortality rates per 100,000 people for both genders and different countries of the world from 1990 to 2019 were extracted from the Global Burden of Disease (GBD) free online database of the Institute for Health Metrics and Evaluation (IHME) of the University of Washington^15^. Then, the mortality-to-incidence ratio (MIR) was calculated by dividing the age-standardized mortality rate by the age-standardized incidence rate for both sexes in each year and in different countries. MIR is a measure of the burden of disease in a specific region or country. This ratio determines whether a country or region has a higher or lower mortality rate for a specific disease that is normalized to its incidence. MIR is also a simple and common method to estimate the 5-year relative survival rate of patients^16,17^.

Human Development Index (HDI) data were also from the United Nations Development Program (UNDP) database for each country from 1990 to 2021^18^. Accordingly, the countries with HDI values less than 0.788 were considered developing countries, and countries with HDI values of 0.788 or higher were taken as developed countries^19^. Finally, 189 countries with available incidence and mortality rates and HDI data were included in the study.

### Statistical analysis

In this longitudinal study, summary statistics related to DM incidence rate, mortality rate, and MIR were presented for each IHME region from 1990 to 2019. Then, the annual mean trends of the indicators were graphically shown based on the region and the development level. In the next step, the marginal modeling approach and the Generalized Estimating Equation (GEE) method were used to evaluate the longitudinal effect of development on the incidence, mortality, and MIR of DM. GEE is a population-level approach that allows researchers to obtain estimates of model parameters that are averaged over the entire population^20^. To examine the mean trends in DM burden indices, the marginal model was fitted separately for developed and developing countries as shown in Eq. (1):

1 $${\mu }_{ij}={\beta }_{0}+{\beta }_{1}{time}_{ij}$$

where ${\mu }_{ij}$ is the mean indexes for country i (i = 1,2,…,189) in year j (j = 1,2,…,30), ${\beta }_{0}$ is the intercept, and ${\beta }_{1}$ is the slope of the model showing the mean annual changes in the indices. Moreover, for cases where the mean trend had a non-linear pattern, the spline model (Eq. (2)) was used:

2 $${\mu }_{ij}={\beta }_{0}+{\beta }_{1}{time}_{ij}+{{\beta }_{2}{(time}_{ij}-{t}^{*})}_{+} \left\{\begin{array}{ll}{{(time}_{ij}-{t}^{*})}_{+}=0, &\quad { time}_{ij}\le {t}^{*}\\ {{(time}_{ij}-{t}^{*})}_{+}={time}_{ij}-{t}^{*},&\quad {time}_{ij}>{t}^{*}\end{array}\right.$$

where ${t}^{*}$ is the turning point of the mean trend graph^21^. All statistical procedures and model fitting were performed in SPSS-26 and STATA-17 software.

## Results

**Figure 1:**
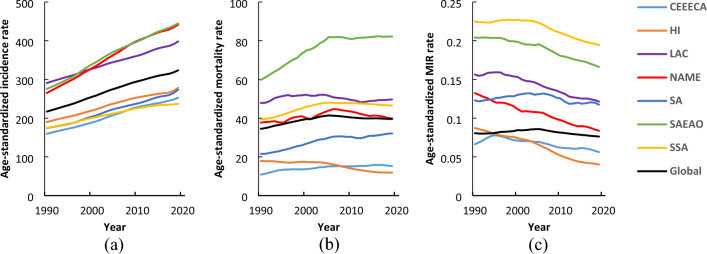
Mean trends of DM (**a**) incidence, (**b**) mortality, and (**c**) MIR rates per 100,000 individuals by IHME super regions in the period 1990–2019.

A total of 189 countries were included in the study to evaluate the DM incidence, mortality, and MIR from 1990 to 2019. Figure 1 shows the trends of the DM incidence, mortality, and MIR in different regions based on the IHME data. As can be seen, all regions experienced a significant increase in the incidence of DM during the 30 years (Fig. 1a). The mortality rate for LAC was relatively stable during the period and the HI region experienced a downward trend in the mortality rate. Moreover, the CEEECA, NAME, SA, SSA, and SAEAO regions showed an upward trend in the mortality rate due to DM. However, the NAME countries had an upward trend from 1990 to 2006 and a decreasing trend in their DM mortality from 2006 to 2019. In addition, the SAEAO countries experienced a significant upward trend from 1990 to 2005, but they followed a steady trend from 2005 to 2019. Likewise, SSA displayed an increasing trend from 1990 to 2004 and followed a stable trend from 2004 to 2019 (Fig. 1b). The trend of diabetes-induced MIR for the CEEECA and SA regions was relatively stable over the period, while an upward pattern was observed in other regions (Fig. 1c).

Table 1 shows the descriptive statistics for DM incidence, mortality, and MIR in each IHME region and all countries studied in 5-year intervals and 2019. As shown in the table, the countries in the SAEAO region have the highest incidence and mortality rates and the countries in the SSA region have the highest MIR from 1990 to 2019. In addition, the maximum incidence and mortality rate were found in the SAEAO region in 2019, and the maximum MIR was observed in the SSA region in 1990.

**Table 1:** Mean trend of DM incidence, mortality, and MIR per 100,000 by IHME super region over 1990–2019.

Super region	Index	Year						
		1990	1995	2000	2005	2010	2015	2019
CEEECA (29 countries)	Incidence	159.6 (48.2)^a^	173.3 (52.8)	188.7 (54.6)	209 (62.5)	228.6 (69.9)	239.6 (80.4)	252.7 (85.4)
	Mortality	10.8 (5.2)	13.4 (6.7)	13.7 (8.3)	15.1 (10.4)	15.1 (11.4)	15.9 (12)	15.2 (11.2)
	MIR	0.07 (0.02)	0.08 (0.03)	0.07 (0.04)	0.07 (0.04)	0.06 (0.04)	0.06 (0.04)	0.06 (0.03)
HI (34 countries)	Incidence	190.1 (90.4)	204.1 (97.3)	219.7 (99)	238.3 (104.3)	253.5 (102.4)	263.6 (94.4)	278.1 (89.2)
	Mortality	17.9 (19.9)	17.2 (17.3)	17.3 (17.9)	16.5 (17.8)	13.9 (15.4)	12.3 (13.8)	11.9 (12.5)
	MIR	0.09 (0.04)	0.08 (0.04)	0.07 (0.04)	0.07 (0.04)	0.05 (0.03)	0.04 (0.03)	0.04 (0.02)
LAC (30 countries)	Incidence	290.8 (87.5)	308.2 (90.6)	327.4 (92.9)	345.7 (91.5)	361 (93.2)	381.5 (94)	398.1 (94.9)
	Mortality	47.8 (29.6)	51.5 (29.8)	52.1 (30.1)	51.6 (28)	49.3 (24.8)	48.9 (23.6)	49.7 (24.2)
	MIR	0.16 (0.07)	0.16 (0.06)	0.15 (0.06)	0.14 (0.06)	0.13 (0.05)	0.13 (0.05)	0.12 (0.04)
NAME (21 countries)	Incidence	265 (93.8)	295.5 (118.6)	329.2 (140.8)	365.1 (154.8)	399.7 (154.1)	422.7 (153.1)	441.5 (143.4)
	Mortality	37.7 (27.6)	37.7 (27.7)	40.1 (33.5)	43.9 (42.2)	43.4 (41.3)	41.3 (36.7)	39.8 (32.9)
	MIR	0.13 (0.06)	0.12 (0.06)	0.11 (0.05)	0.11 (0.06)	0.10 (0.05)	0.09 (0.05)	0.08 (0.04)
SA (5 countries)	Incidence	174.4 (20.6)	185.4 (23.2)	204 (22.3)	224 (29.3)	237.8 (34.3)	255.6 (37.6)	273.3 (38.1)
	Mortality	21.6 (6.3)	23.5 (7.7)	26.8 (10)	29.9 (12.6)	30.4 (12.5)	30.9 (12.1)	32.1 (11.7)
	MIR	0.12 (0.03)	0.13 (0.04)	0.13 (0.04)	0.13 (0.05)	0.13 (0.04)	0.12 (0.03)	0.12 (0.04)
SAEAO (25 countries)	Incidence	275.7 (100.7)	302.4 (117.4)	339.1 (139.7)	372.5 (156.3)	397.9 (165.4)	425.2 (168.7)	445 (169.3)
	Mortality	59.8 (38.8)	67.0 (51.7)	74.9 (62.9)	81.8 (67.5)	80.8 (64.8)	82 (64.2)	82.2 (64.1)
	MIR	0.20 (0.08)	0.20 (0.09)	0.20 (0.09)	0.20 (0.10)	0.18 (0.09)	0.17 (0.09)	0.17 (0.08)
SSA (45 countries)	Incidence	174.6 (37.4)	186 (40.5)	200.7 (46.1)	212.8 (51.8)	225.5 (58.7)	233.8 (60.7)	237 (56.5)
	Mortality	39.3 (13.7)	41.9 (14.7)	45.9 (18.9)	47.9 (23.7)	47.8 (23.5)	47.2 (22.7)	46.5 (21.3)
	MIR	0.22 (0.05)	0.23 (0.05)	0.23 (0.06)	0.22 (0.06)	0.21 (0.06)	0.20 (0.05)	0.19 (0.05)
Global (189 countries)	Incidence	216.9 (90.7)	234.3 (101.3)	255 (112.6)	276.3 (122.4)	295 (127.8)	310.4 (132.4)	323.6 (133.8)
	Mortality	34.5 (28.1)	37 (31.5)	39.5 (36.2)	41.4 (39.6)	40.4 (38.5)	39.9 (37.8)	39.6 (37.2)
	MIR	0.15 (0.08)	0.15 (0.08)	0.14 (0.08)	0.14 (0.09)	0.13 (0.08)	0.12 (0.08)	0.12 (0.08)

*CEEECA* Central Europe, Eastern Europe, and Central Asia, *HI* high income, *LAC* Latin America and Caribbean, *NAME* North Africa and Middle East, *SA* South Asia, *SAEAO* Southeast Asia, East Asia, and Oceania, *SSA* Sub-Saharan Africa.

^a^Mean (standard deviation).

Table 2 shows the descriptive statistics for DM indicators by developing and developed countries in the studied period. As can be seen, developing countries had a lower incidence rate and a higher mortality rate and MIR in the period in question.

**Table 2:** Mean trend of DM incidence, mortality, and MIR per 100,000 by development factor over 1990–2019.

Countries	Index	Year						
		1990	1995	2000	2005	2010	2015	2019
Developing (118 countries)	Incidence	215.9 (85.1)^a^	233.1 (94.1)	254.6 (105.5)	275.4 (113.7)	294.4 (119.9)	310.6 (126.2)	323.3 (130.7)
	Mortality	40.2 (27.3)	43.9 (32.3)	47.7 (37.9)	50.1 (40.5)	49.4 (38.5)	49.9 (38.4)	49.8 (38.2)
	MIR	0.18 (0.08)	0.18 (0.08)	0.18 (0.08)	0.18 (0.08)	0.16 (0.07)	0.16 (0.07)	0.15 (0.07)
Developed (71 countries)	Incidence	218.6 (100)	236.3 (113)	255.7 (124.3)	277.7 (136.4)	296.1 (140.7)	310 (143.1)	324.1 (139.8)
	Mortality	25 (27)	25.4 (26.6)	25.8 (28.6)	26.9 (33.4)	25.3 (33.6)	23.2 (30.2)	22.5 (28.4)
	MIR	0.10 (0.06)	0.09 (0.05)	0.09 (0.05)	0.08 (0.05)	0.07 (0.05)	0.06 (0.05)	0.06 (0.04)

^a^Mean (standard deviation).

**Figure 2:**
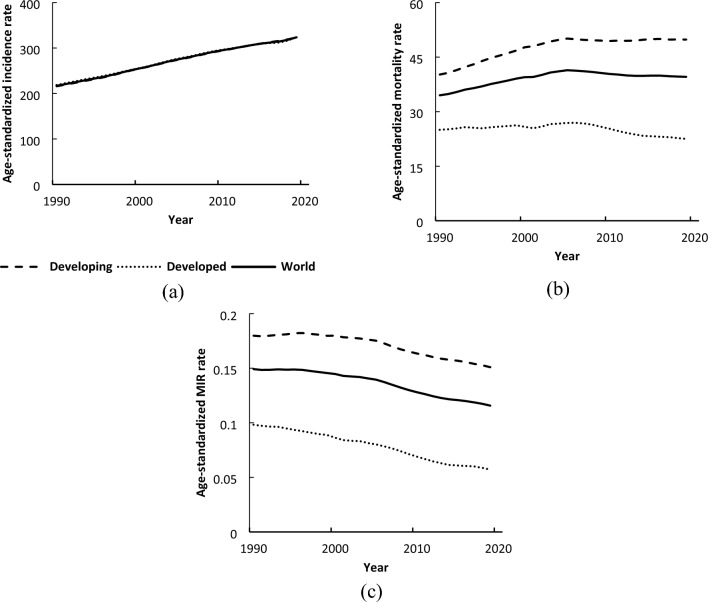
Mean trends of DM (**a**) incidence, (**b**) mortality, and (**c**) MIR rates per 100,000 individuals by development factor and total world countries in the period 1990–2019.

Figure 2 displays the trends of DM incidence, mortality, and MIR for developing countries, developed countries, and the whole world from 1990 to 2019. As shown in this figure, the mean incidence and MIR for developing, developed countries, and the whole world seem to follow a linear trend. Thus, the linear GEE model was used to assess the mean trends. Moreover, as the mean mortality rate for all three regions follows a non-linear pattern, the spline model (the existence of a peak in 2005) was used to investigate the DM mortality trends.

The estimated coefficients and parameters based on the GEE and spline model are presented in Table 3. The results in this table suggest that the developed countries had an intercept of about 3 per 100,000 people more than the developing countries. This means that the mean incidence rate in developed countries was about 3 per 100,000 people higher than the incidence of diabetics in developing countries in 1990. In addition, the mean incidence rates in developed and developing countries follow an upward trend, and the estimated slope of the mean incidence rates is almost the same (3.65 vs. 3.78), indicating a relatively similar increase in the mean incidence of diabetics in both developing and developing countries. Moreover, the estimated intercept for the incidence of DM in all countries was about 213.96 per 100,000 people in 1990 with a positive slope with an annual increase of 3.73 per 100,000 from 1990 to 2019 for the entire world.

**Table 3:** Parameter estimates from modeling the mean trend of DM incidence, mortality, and MIR by development factor between 1990 and 2019.

HDI level	Index	Parameter	Estimate	Standard error	P-value
Developing	Incidence	Intercept	212.67	8.27	< 0.001
		Time	3.78	0.21	< 0.001
	Mortality (non-linear)	Intercept	40.13	1.67	< 0.001
		Time	0.62	0.15	< 0.001
		(Time-16)	− 0.64	0.28	0.023
	MIR	Intercept	0.188	0.0074	< 0.001
		Time	− 0.001	0.0001	< 0.001
Developed	Incidence	Intercept	216.10	12.94	< 0.001
		Time	3.65	0.31	< 0.001
	Mortality (non-linear)	Intercept	24.88	1.77	< 0.001
		Time	0.12	0.16	0.459
		(Time-16)	− 0.45	0.30	0.135
	MIR	Intercept	0.103	0.0070	< 0.001
		Time	− 0.002	0.0001	< 0.001
Total World	Incidence	Intercept	213.96	7.09	< 0.001
		Time	3.73	0.18	< 0.001
	Mortality (non-linear)	Intercept	34.40	1.30	< 0.001
		Time	0.43	0.12	< 0.001
		(Time-16)	− 0.57	0.22	0.010
	MIR	Intercept	0.16	0.0061	< 0.001
		Time	− 0.001	0.0001	< 0.001

*HDI* human development index.

The intercept for the mortality rate in developing countries was about 15 per 100,000 people more than in developed countries. The mean mortality rate from DM in developing countries increased annually until 2005 with a positive slope of 0.62 per 100,000 people and then followed a downward trend with a slope of 0.02 from 2005 to 2019. The mean trend mortality rate for developed countries was almost constant during the study period (P > 0.05). The mean mortality rate for all countries shows that the estimated intercept for the mortality rate in all countries in 1990 was about 34.40 per 100,000 people and then the annual mean mortality rate for DM increased with a positive slope of 0.43 per 100,000 people until 2005 and then followed a downward trend with a slope of 0.14 from 2005 to 2019.

Finally, the mean MIR for developing countries was about 1.8 times that of developed countries in 1990. Likewise, the estimated slope for the mean MIR in developed countries followed a downward trend and was twice that of developing countries from 1990 to 2019. Moreover, the mean global MIR decreased significantly with an annual decrease of about 0.001 units.

## Discussion

The results of this study showed that the global mean incidence rate of DM followed an upward trend in both developed and developing countries from 1990 to 2019. The global mean mortality rate due to DM also followed an upward trend in developing countries until 2005, and then a downward trend, but showing no significant change in developed countries over time. The mean MIR showed a downward trend in both developed and developing countries, while developed countries had a relatively faster decrease in MIR than developing countries.

An analysis of the global incidence rate of diabetics showed that almost all countries experienced a significant upward trend from 1990 to 2019 with an annual increase of 3.73 per 100,000 people. The upward trend in the incidence of DM was consistent with some other reports around the world. For example, Lin et al. found that the incidence of DM increased globally from 1990 to 2017^14^.

A comparison of the incidence of DM in developed and developing countries from 1990 to 2019 showed no significant difference in the increase in the incidence rate of diabetics in developed and developing countries (slope 3.65 vs. 3.78). Moreover, the descriptive analysis indicated the incidence of DM in developed countries has increased by 48.2% in these 30 years, while this increase in developing countries in the same period has been slightly higher (49.7%). According to Jinli Liu et al., the incidence of DM has increased in both developing and developed countries^13^.

The increased incidence of DM can typically be attributed to a higher prevalence of risky behaviors such as obesity, inactivity, and unhealthy diet. Accordingly, a systematic review study showed that lifestyle interventions such as physical activity and healthy diets can lead to a reduction in DM^22^. Studies have also shown that the prevalence of obesity^23^ and the ratio of energy received through fat has increased over time^24^. In addition, population aging can also be one of the reasons for the increase in the incidence of DM. Studies have confirmed that the incidence of non-communicable diseases increases with population aging^25^. Another factor affecting the increased incidence of DM is the improvement of DM diagnosis methods. With the improvement of DM screening techniques and improved public awareness, people's participation in screening programs has increased, leading to the detection of more diabetic cases. Furthermore, studies have indicated that the percentage of people with undiagnosed DM has decreased over time^26^. A study showed that the number of people who tested HbA1c increased over time. The level of public literacy has also increased in communities^25^. In addition to the more accurate diagnosis methods, measurement and diagnosis methods also adjusted, for example, screening criteria have changed over time and the cut-off points for DM diagnosis have decreased over time^27^.

An analysis of the DM mortality rate for each IHME region indicated that all regions except HI have had an increasing trend in the DM mortality rate from 1990 to 2019, as confirmed in some other reports around the world. For example, Lin et al. found that the global trend of mortality due to DM increased from 1990 to 2017^14^. Other studies have also reported a downward trend in DM mortality in the countries in the HI region, including Canada^28^, Australia^29^, United Kingdom, and several European countries^30^. According to Xiling Lin et al., the mortality rate of DM in high-income countries decreased from 1990 to 2017^14^.

The data in the present study also showed the global mean mortality rate due to DM increased by 0.43 per 100,000 people annually until 2005 following a decrease by a factor of 0.14 in each follow-up year. One of the reasons for the reduction of mortality due to DM can be the reduction of complications due to DM. Accordingly, Mohammed K Ali et al., found that hospital admissions due to diabetic complications decreased after 2005^31^.

Our findings also suggested that the DM mortality rate in developing countries has increased by 23.9% in 30 years but decreased by 9.9% in developed countries. In a similar vein, Xiling Lin et al. compared the mortality rates due to DM between developing and developed countries and reported that the number of death due to DM increased in developing countries annually until 2005 with a positive slope of 0.62 in every 100,000 people and then followed a downward trend with a negative slope of 0.02. However, no significant change was observed in developed countries over time. The decreased mortality rate caused by DM in developing countries can be attributed to the developments in DM education, continuous monitoring of blood sugar, and widespread use of insulin and its analogs^32,33^. The absence of a significant change in DM mortality in developed countries can be attributed to improvements in developed countries in previous years. However, the mortality rate has followed a stable trend since then. In contrast, a study by Lin et al. and the report of the World Health Organization showed that the mortality rate due to DM has increased in the world^14^. A reason for such contradictory findings is that previous studies in the literature have used only descriptive statistics, while robust analytical techniques such as GEE and spline model were used in the present study to assess the linear and non-linear indicators of the DM incidence, mortality, and MIR.

In the present study, MIR was used as a surrogate index for the five-year survival rate of diabetic patients. According to our findings, MIR followed a downward trend from 0.15 in 1990 to 0.12 in 2019 with a partial slope of 0.001 (showing an mean annual decrease). Consistent with the findings of the present study, a meta-analysis study showed that the mortality proportion among DM patients in the world decreased by 43% from 1970 to 1989, by 53% from 1990 to 1999, and by 74% from 2000 to 2016^34^. MIR is a measure of the number of patients who die after developing DM. This index can indicate the improvement of early diagnosis and better control of the disease^35^. A look at the MIR trend in IHME regions shows that HI countries have had the largest decrease (from 0.09 to 0.04 with a 54% decrease) in these years, while the countries in the SA region have had the lowest decrease (from 0.123 to 0.117 with a 5% decrease) in the same period. Moreover, during a 30-year follow-up, developed countries had a greater reduction in MIR of DM than developing countries. It seems that the survival rate of patients in the richest region has improved faster compared to other regions. The key reasons for this increase in survival rates are likely to be more widespread screening programs and early detection of the disease, the promotion of knowledge, attitudes, and practices of people around the world about DM prevention strategies, and improved levels of care for diabetic patients.

The significant association between HDI and MIR shows that in recent years, developed countries have focused on improving the adoption of a healthy lifestyle as well as access to health care services in the prevention or control of DM and increasing life expectancy. On the other hand, socioeconomic status is directly associated with the patient's survival. Thus, patients with higher socioeconomic status may experience more survival due to greater and better access to DM related care and services^36^.

The most important limitation in the present study was the unavailability of accurate and reliable data on DM incidence and mortality rates in some countries, especially in less developed areas, is a significant limitation that may affect the accuracy of the study's findings and conclusions. Therefore, future studies should focus on collecting more accurate and reliable data on DM incidence and mortality. However, the main strength of the present study was the use of longitudinal GBD data with a long follow-up period with 30 repeated measurements and the use of advanced statistical models that enabled us to obtain more accurate estimates. On the other hand, using MIR as a proxy for survival is a reliable approach to explain the differences in the age-standardized incidence and mortality rates due to DM in different geographical areas. To calculate this index, there is no need to conduct studies with long-term follow-up, which are potentially time-consuming, expensive, and prone to various biases. Moreover, to the best of our knowledge, unlike the present study, no study has evaluated the relationship between development and MIR of DM.

## Conclusion

The results in the present study indicated that the increased incidence of DM is one of the most important health concerns in the world. With the extensive changes in recent years in people's lifestyles, increasing urbanization, industrialization, and population aging in developing countries, the incidence and mortality rates of diabetics are somewhat similar to developed countries. If the changes in the risk factors for DM are not taken into account, this disease can cause more concerns for communities in the coming years. This being so, countries should implement cost-effective DM prevention and control programs to increase public awareness of DM risk factors, promote active lifestyles, improve nutrition, and increase access to diagnostic and treatment services.

## Data Availability

The datasets generated and analysed during the current study are available from the corresponding author upon reasonable request.

## Abbreviations

DM: Diabetes mellitus
MIR: Mortality-to-incidence ratio
GBD: Global burden of disease
HDI: Human development index
UNDP: United nations development program
GEE: Generalized estimating equations
WHO: World health organization
ADA: American diabetes association
IHME: Institute for health metrics and evaluation
CEEECA: Central Europe, Eastern Europe, and Central Asia
HI: High income
LAC: Latin America and Caribbean
NAME: North Africa and Middle East
SA: South Asia
SAEAO: Southeast Asia, East Asia, and Oceania
SSA: Sub-Saharan Africa
